# The effectiveness of interventions to reduce peripheral blood culture contamination in acute care: a systematic review protocol

**DOI:** 10.1186/s13643-018-0877-4

**Published:** 2018-11-30

**Authors:** J. A. Hughes, C. J. Cabilan, Julian Williams, Mercedes Ray, Fiona Coyer

**Affiliations:** 1 School of Nursing, Queensland University of Technology, Brisbane, Australia; 2 Emergency and Trauma Centre, Royal Brisbane and Women’s Hospital, Ground Floor, Dr James Mayne Building, Butterfield Street, Herston, Brisbane, Queensland 4029 Australia; 3 Clinical Research Officer, Emergency Department, Princess Alexandra Hospital, 199 Ipswich Road, Woolloongabba, Brisbane, Australia; 4 Faculty of Medicine, University of Queensland, Brisbane, Australia; 5 Intensive Care Services, Critical Care and Clinical Support Services, Royal Brisbane and Women’s Hospital, Brisbane, Australia; 6 Institute for Skin Integrity and Infection Prevention, University of Huddersfield, Huddersfield, UK

**Keywords:** Systematic review, Blood culture, Contamination, Microbial contamination, Acute care, Peripheral blood collection

## Abstract

**Background:**

Blood cultures are an integral part of the diagnosis of bacteremia in unwell patients. The treatment of bacteremia involves the rapid and accurate identification of the causative agent grown from the blood cultures collected. Contamination of blood cultures with non-pathogenic microbes such as skin commensals causes false positive results and subsequent unnecessary and potentially harmful interventions. While guidelines for blood culture quality recommend no more than 2–3% contamination rate, rates up to 12% are reported in the literature. There have been a number of methods proposed to reduce the contamination of blood cultures, including educational interventions, changing of skin cleansing preparations and introduction of blood culture collection packs in acute care settings. This protocol outlines methods to identify and evaluate interventions to reduce blood culture contamination in the acute care setting.

**Methods:**

The reviewers will conduct a systematic search of literature in CINHAL, PubMed, EMBASE and the Cochrane *Central* register of controlled trials. Unpublished works will be identified in ProQuest Dissertations and Theses. Articles will be assessed for relevance based on their title and abstract. Remaining relevant citations will have their full text retrieved and assessed against eligibility criteria. All studies that meet the eligibility criteria will have their methodological quality appraised. Assessments for relevance and methodological quality will be conducted independently by two reviewers. If appropriate, data will be analysed using the Mantel-Haenszel method under a random effects model. Heterogeneity of the studies will be assessed using the *I*^2^ and chi-squared statistic. Meta-analysis will be attempted if the data is suitable.

**Discussion:**

This review will identify and summarise the interventions previously described in the literature aimed at reducing peripherally collected blood culture contamination rates in acute care. These findings have the potential to lead to multifaceted interventions based on previous evidence to reduce blood culture contamination in the acute setting. Reductions in the proportion of contaminated blood cultures have the potential to save money, unrequired treatment (particularly antimicrobials) and hospital bed days.

**Systematic review registration:**

In accordance with guidelines outlined in the PRISMA-P methodology, this protocol was registered with the International Prospective Register of Systematic Reviews (PROSPERO) on December 8, 2017, and last updated on January 4, 2018 (registration number CRD42017081650).

## Background

A blood culture (BC) is a test designed to detect viable bacteria or fungi in the bloodstream. A sample of blood obtained using a sterile technique is placed in a culture media and incubated in a controlled environment, usually between 1 and 7 days [1, 2]. Blood can be collected by a number of different methods, but is usually collected from a peripheral vein by direct venipuncture or drawn from an existing invasive device such as a peripheral cannula or central venous access device. Both methods have the opportunity to introduce microorganisms that are not present in the blood into the blood sample. Outside of intensive care and oncology treatment areas, blood for culture is most commonly collected from a peripheral vein, either via dedicated venepuncture or through an existing peripheral intravenous catheter (PIVC) device. At the bedside, prescribed volumes of collected blood are immediately transferred to bottles containing aerobic and anaerobic culture media.

Blood cultures are an essential diagnostic tool for the identification and treatment of bacteremia. Bacteremia is a significant source of morbidity and mortality, with up to 37% mortality reported [3]. The treatment of bacteremia requires the rapid and accurate identification of the causative organism [4]. As for any test or procedure conducted in healthcare, the quality of the results from the analysis is directly attributable to the quality of the process in which the procedure was carried out. Blood cultures are not immune to variations in quality secondary to variations in collection techniques. Contamination of BC with organisms not originating from the blood of the patient is common. Current guidelines recommend that institutions should aim for a maximum of 2–3% contamination rate for BC collected [5–7]. Other guidelines recommend testing from at least two peripheral culture sites with at least 7 mls of blood in each culture bottle (both aerobic and anaerobic) [3].

Contamination rates in BC vary in the literature, with reported figures between 3 and 12% [7–13]. However, most baseline contamination rates reported are above the 3% recommendation in the current guideline [5]. Some of the variation in reported contamination rates may relate to differing criteria used to define contaminant organisms. The Clinical and Laboratory Standards Institute defines contamination as a “microorganism isolated from a blood culture during specimen collection or processing [and was] not pathogenic for the patient from whom the blood was collected” [2,p.2]. Some authors specify microorganisms that are considered contaminants [8–10], while other authors refer decisions regarding contaminant status to contributing microbiologists [11]. Contamination rates provide an important metric on quality of care delivery and should be maintained at the lowest possible rate regardless of the differences in the definitions used.

Blood culture contamination is a significant problem because it increases the incidence of false positive results. Contaminated or false positive BC may have detrimental effects to the patient, the organisation and to antimicrobial stewardship efforts. Contaminated BC may result in unnecessary treatment to the patient [3]. Patients who have a false positive BC have an extended stay in hospital compared to those patients who have a negative BC. In the North American setting, this has been quantified as an additional 4.5-day length of stay for patients with false positive BC compared to those patients with true negative results [3, 10, 12]. In the UK, it has been shown that patients with false positive BC results extend their hospital length of stay by 5.4 days [10]. Numerous studies have identified the financial burden of BC contamination, such as costs of cultures, increased length of stay, and pharmacy costs [3, 7, 10, 12, 13]; however, many of these estimates of cost are based on figures by Bates et al. work from 1991 and may not be relevant to current healthcare expenditure. Contemporary reports (from a district teaching hospital in Ireland) have reported costs $7500 per patient with a false positive blood culture [10]. The collection of contaminated BC also has the unwanted effect of prolonging broad-spectrum antibiotic therapy [3]. The increased use of antibiotics (especially broad—spectrum) for extended periods of time can lead to resistance in the microbes present. Antibiotic resistant bacteria are one of the greatest challenges in healthcare today. With increasing rates of resistance and fewer new antibiotics being manufactured, judicious use of currently available broad-spectrum antibiotics is paramount [14].

Some interventions have been proposed to reduce the number of contaminated BC in the acute care environment. These have included informational responses, such a regular emails outlining the current contamination rates [7, 9, 12, 13, 15, 16], the introduction of chlorhexidine swabs at the point of collection [11, 13, 17] specialised collection packs [8, 12, 15, 18] and individual feedback on rates of contamination and procedural technique [7, 16, 19]. The grouped effectiveness of these interventions is unclear. There are three previous papers that provide aggregated results of interventions to reduce BC contamination. All three of these works have limitations that will be mitigated in this systematic review. One study provided a literature review with limited databases searched and no attempt at meta-analysis [20], another only reviewed skin antiseptic interventions [21] and the third reported on a limited number of interventions [22]. This systematic review will look at all reported interventions aimed at reducing the amount of contaminated peripheral BC in the adult population.

This systematic review will focus on peripherally collected (through venepuncture or from PIVC) BC, as these are the most commonly collected cultures. Collection of BC from invasive devices introduces other opportunities for contamination that are not present in peripherally collected cultures. The main outcome of interest will be reduction in contamination rates of peripherally collected blood cultures, while other outcome measures such as the reduction in antimicrobial usage, time spent in hospital and costs of providing care will be examined if the literature allows.

## Methods/design

### Objectives

The aims of this systematic review are to identify interventions reported in the literature which aimed to reduce contamination from peripherally collected BC and to evaluate the effectiveness of these interventions.

### Inclusion criteria

Studies that meet the following criteria will be included in this systematic review.

#### Study designs

The review will include all studies that assess the effectiveness of intervention/s against a control or usual care. The studies will include randomised controlled trials, quasi-experimental studies, pretest-posttest and time series designs. Studies that do not have a control or comparator group will not be considered for inclusion.

#### Participants

Studies on all groups of adult acute care patients will be included in this review. Interventions aimed specifically at populations under 16 years old or using paediatric blood culture collection bottles will be excluded. The use of paediatric blood culture bottles typically uses one and not two bottles; therefore, there is less chance of contamination. Patients under the age of 16 years may have different contamination risk compared to adults and therefore should be studied separately. If sufficient evidence is found then subgroup analysis may be used to identify differences in settings (i.e. emergency department vs intensive care unit interventions or general wards).

#### Interventions

All interventions aimed at improving the quality of peripherally collected blood cultures described in the eligible studies will be included in this review. The quality of peripherally collected blood cultures is measured in terms of contamination, single sets of cultures and volume of blood cultured. The interventions can include, but not be limited to changes in practice or procedure, education interventions, product or device interventions, or a combination of interventions.

#### Comparators

The comparator will be control, usual or standard care, or no intervention.

#### Outcomes

The primary outcome will be the percentage of blood culture sets (defined by paired aerobic and anaerobic culture bottles) in which a contaminant was isolated. The Clinical and Laboratory Standards Institute defines contamination as, “A micro-organism isolated from a blood culture that was introduced into the culture during specimen collection or processing that was not pathogenic for the patient from whom the blood was collected” [2]. However, for the purposes of this review, the definition of contaminant will necessarily vary between included studies. Other outcomes will also be considered and may include length of stay, antibiotic therapy used in terms of type, frequency and dosage, and additional costs incurred in the delivery of care, such as increases in length of stay, antimicrobal therapy and associated costs.

#### Setting

Studies that were conducted in hospital settings will be included. The scope of the review is within acute care settings so studies that were conducted in general practice, commercial laboratory collection centres, residential aged care facilities or research facilities will be excluded.

#### Language

Due to unavailability of funding for language translation, the review will only include studies that are published in or have been previously translated into English.

### Search strategy

The search strategy will be exhaustive to include published and grey literature. No limitations will be placed on the date of the conduct or publication of the study. Due to a direct lack of funding for translation, the review will only include studies that are published in or have been previously translated into English. Published literature will be sourced in Cumulative Index of Nursing and Allied Health Literature (CINAHL via Ebsco) from 1981 to present, PubMed from 1949 to current, Excerpta Medica database (EMBASE) from 1947 to current and the Cochrane Central Register of Controlled Trials (CENTRAL) from 1996 to current. Unpublished studies will be searched for in ProQuest Dissertations and Theses from 1743 to current. An example of a search strategy for CINAHL and PubMed databases are detailed below in Table 1. Searches will also be conducted in registeries of systematic reviews such as the International Prospective Register of Systematic Reviews (PROSPERO) and in registeries of clinicial trials (Australia and New Zealand Clinical Trials Register, International Clinical Trials Registery Platform Search Portal and ClinicalTrials.gov). In addition to searches of the abovementioned databases, the reviewers will hand search the reference lists of relevant literature. The following are examples of the search strategies used for PubMed and CINAHL databases:

**Table 1:** Examples of search strings and results from PubMed and CINAHL via EbscoHost

Keyword search	Date of search	Search engine used	Number of publications retrieved
((((contamination[Title/Abstract]) OR false-positive[Title/Abstract])) AND ((((Blood culture[MeSH Major Topic]) OR Hematologic tests[MeSH Major Topic]) OR blood culture*[Title/Abstract])	November 28, 2017	PubMed	1491
(Blood Culture OR Culture) AND (Contamination OR False Positive OR Microbial Contamination OR Bacterial Contamination OR False Negative).	November 29, 2017	CINAHL via Ebsco	2119

### Selection of studies

Selection will be conducted independently by two reviewers (JH and CJC). Any disagreement will be resolved with referral to a third reviewer whose decision will be final (FC). All citations from the final search strategy will be imported into Endnote and subsequently screened for relevance using title and abstract. The full text of relevant citations will be retrieved and assessed for eligibility against the inclusion criteria set above. All eligible studies will be appraised for methodological quality using the Effective Practice and Organisation of Care group risk of bias criteria (Tables 2 and 3) [23].

**Table 2:** Effective Practice and Organisation of Care group risk of bias criteria for randomised controlled trials, quasi-experimental studies and pretest-posttest designs

	High risk	Low risk	Unclear risk
1. Was the allocation sequence adequately generated?	□	□	□
2. Was the allocation adequately concealed?	□	□	□
3. Were baseline outcome measurements similar?	□	□	□
4. Were baseline characteristics similar?	□	□	□
5. Were incomplete outcome data adequately addressed?	□	□	□
6. Was knowledge of the allocated interventions adequately prevented during the study?	□	□	□
7. Was the study adequately protected against contamination?	□	□	□
8. Was the study free from selective outcome reporting?	□	□	□
9. Was the study free from other risks of bias?	□	□	□

**Table 3:** Effective Practice and Organisation of Care group risk of bias criteria for interrupted time series

	High risk	Low risk	Unclear risk
1. Was the intervention independent of other changes?	□	□	□
2. Was the shape of the intervention effect pre-specified?	□	□	□
3. Was the intervention unlikely to affect data collection?	□	□	□
4. Was knowledge of the allocated interventions adequately prevented during the study?	□	□	□
5. Were incomplete outcome data adequately addressed?	□	□	□
6. Was the study free from selective outcome reporting?	□	□	□
7. Was the study free from other risks of bias?	□	□	□

### Data extraction

Data extraction will be conducted independently by two reviewers (JH and CJC). It is anticipated that some publications will have missing or incomplete data. In those instances, the corresponding authors will be contacted. If authors do not respond after being contacted three times and data are presented graphically, the WebPlotDigitizer [24] on-line application will be used to quantify data. Data will be entered into Review Manager 5.3.5 software [25]. Meta-analysis will be conducted where possible, otherwise findings will be presented in a narrative form.

Events (e.g. contamination, antibiotic therapy) will be analysed using Mantel-Haenszel method under the random effects model and presented as risk ratio (RR) [26]. A RR of > 1 will be interpreted as increased likelihood of the event, while decreased chance of the event occurring will have corresponding value of < 1. A RR with a 95% confidence internal (CI) that does not include the value of 1 will be considered statistically significant [27]. Continuous data such as cost, time, and length of stay will be presented as mean difference and will be analysed using random effects inverse variance. In addition to these data other information such as participant charcteristics, details of the intervention, sustainability, funding sources, and location of intervention will be captured and summarised. Grading of the quality of the evidence and the strength of any conclusion made from this analysis will be conducted using the Grading of Recommendations Assessment, Development and Evaluation (GRADE) criteria [28].

### Assessment of heterogeneity

Heterogeneity will be assessed statistically using the standard *I*^2^ statistic, where (approximately) 1 to 25%, 26 to 75% and 76 to 100% will be interpreted as low, moderate, and high respectively [29]. Moreover, the chi-squared statistic will also be used to determine heterogeneity, where a *p* value of less than 0.1 will indicate significant heterogeneity. If present, heterogeneity will be explored in a sensitivity analysis accounting for population characteristics, setting, effect size, or sample size.

## Discussion

Blood culture contamination is a source of error in healthcare. False positive BCs are associated with unnecessary and potentially harmful interventions. This is associated with increased costs of healthcare, and inappropriate antibiotic usage. A number of interventions have previously been trialled to reduce the amount of BC contamination in acute care. This systematic review will identify the interventions reported in the literature that have previously been trialled in an attempt to reduce the BC contamination rates and report on their success with, where possible, pooled results. The identification of interventions that reduce BC contamination in the acute care setting has the potential to influence care. This study will be limited to the adult acute care population; therefore, interventions and influences on paediatric patients and patients in other settings will not be examined, and no inference to these populations should be made. There is the possibility that some evidence that is not reported in English may be missed. However, this represents a minimal amount of the total evidence. The results of this systematic review should be seen as a guideline for the interventions required in acute care to reduce the contamination rate of BC and ultimately improve care.

## Abbreviations

BC: Blood culture
GRADE: Grading of Recommendations Assessment, Development and Evaluation
MeSH: Medical Subject Headings
PIVC: Peripheral intravenous catheter
RR: Risk ratio
